# Gastrin-Releasing Peptide (GRP) Stimulates Osteoclastogenesis in Periodontitis

**DOI:** 10.3390/cells10010050

**Published:** 2020-12-31

**Authors:** YunJeong Choi, Soon Chul Heo, Yu Na Kim, Ji-Young Joo, Jae Joon Hwang, Moon-Kyoung Bae, Hyung Joon Kim

**Affiliations:** 1 Department of Oral Physiology, Periodontal Diseases Signaling Network Research Center, and Dental and Life Science Institute, School of Dentistry, Pusan National University, Yangsan 50612, Korea; celinechoi@pusan.ac.kr (Y.C.); snchlheo@gmail.com (S.C.H.); kyn0394@naver.com (Y.N.K.); 2 Department of Periodontology and Dental Research Institute, Pusan National University Dental Hospital, Yangsan 50612, Korea; joojy@pusan.ac.kr; 3 Department of Oral and Maxillofacial Radiology and Dental Research Institute, Pusan National University, Yangsan 50612, Korea; softdent@pusan.ac.kr

**Keywords:** GRP, osteoclastogenesis, bone resorption, periodontitis

## Abstract

Periodontitis is a chronic inflammatory disease with alveolar bone resorption and subsequent tooth loss as its ultimate outcomes. Gastrin-releasing peptide (GRP) is a neuropeptide with growth-stimulatory and tumorigenic properties, and neuropeptides have previously been suggested to play a role in the complex cascade of chemical activity associated with periodontal inflammation. In this study, GRP treatment enhanced the differentiation of bone marrow-derived macrophages (BMMs) into osteoclasts, and gastrin-releasing peptide receptor (GRPR) antagonists suppressed the pro-osteoclastogenic effect of GRP. *Grpr*-siRNA knockdown resulted in a significantly lower number of osteoclasts formed as compared with the control. Interestingly, gene expression analysis indicated downregulation of *Grp* and *Grpr* expressions in BMMs during osteoclastogenesis. Moreover, ligature-induced periodontitis model in mice and gingival samples from patients with periodontitis displayed increased immunostaining of GRP in the oral epithelium. Subsequently, stimulation of mouse primary epithelial cells (ECs) and HaCaT cells, human epidermal keratinocytes, with lipopolysaccharides (LPS) of *Porphyromonas gingivalis* or live *P. gingivalis* upregulated *Grp* and *Grpr* expressions. Finally, coculture of *P. gingivalis*-stimulated ECs and BMMs using Transwell system revealed that the differentiation of BMMs was induced when subjected to paracrine activation by LPS- as well as live-*P. gingivalis* stimulated ECs. Taken together, our results demonstrate that the pro-osteoclastogenic properties of BMMs may be modulated by GRP produced by ECs in the periodontal microenvironment.

## 1. Introduction

Periodontitis is one of the most prevalent chronic inflammatory diseases initiated by bacteria and progressed by individual’s host inflammatory response to a dysbiotic microbial biofilm on tooth surfaces [1]. Periodontitis is a slowly progressing disease, but the destruction of the supporting tissues around the teeth, gingiva, periodontal ligament, and alveolar bone, is largely irreversible. The ultimate outcome of the disease is the loss or extraction of teeth that are no longer capable of supporting the functional demands, which significantly impacts the general health and quality of life [2,3,4].

Gastrin-releasing peptide (GRP) is a neuropeptide secreted by cells of neural and endocrine origin [5]. GRP is a mammalian homolog of bombesin (BN), a tetradecapeptide originally isolated from frog skin [6] and a part of the bombesin-like peptide (BLP) family that binds to the same receptor of the amphibian bombesin peptide [7].

Four BLP binding receptor subtypes have been isolated so far. Receptor subtype 1 binds neuromedin B and receptor subtype 2, termed GRPR, binds BN and GRP with high affinity. Receptor subtype 3 is an orphan receptor whose ligand has not been identified yet, and subtype 4 binds BN and GRP with high and low affinities, respectively [5]. GRPR is the most widely expressed BLP-binding receptor subtype in human tissues.

Numerous studies have reported the expression of GRPR in inflammatory diseases and tumors in recent years [5,8]. GRPR is overexpressed in various types of cancer cells, and the production of GRP together with GRPR overexpression result in autocrine tumor growth stimulation [8]. In inflammatory diseases, GRP induces the release of proinflammatory cytokines [9,10]. Moreover, GRP has been associated with increased proliferation and migration of rat vascular smooth muscle cell (VSMCs), which are known as critical steps in the pathogenesis of intimal hyperplasia in atherosclerosis [10]. On the basis of evidence pointing out that there is a bidirectional communication between the vasculature and bone conveyed by cellular, endocrine, and metabolic messengers critical in the maintenance of bone health and vasculature health [11], we set out to examine the effect of GRP on the osteoclastogenesis of bone marrow-derived macrophages (BMMs) and to characterize in detail its role in periodontitis. We hypothesized that GRP may be stimulating osteoclastogenesis, since Pi-induced VSMC calcification was associated with elevated *Grp* and *Grpr* expressions [10]. To test this hypothesis, we treated BMMs with GRP or GRPR antagonists to investigate their effects on osteoclastogenesis. Moreover, we established periodontitis model in mouse and obtained patient samples in order to compare the immunostaining of GRP between the oral epithelium of diseased and healthy groups. Furthermore, we applied a non-contact coculture model via Transwell inserts to study the paracrine signaling between BMMs and ECs in the periodontal microenvironment.

## 2. Materials and Methods

### 2.1. Animals

Mice were purchased from Orient Co., Ltd. (Gapyeong, Republic of Korea). Eleven mice were used as donors of BMMs and ECs. At least three mice were analyzed for the periodontitis model. Randomization was used to allocate periodontitis and control groups. Blinded outcome assessment and the data analysis were performed. All animals were maintained under a 12 h light-dark cycle with ad libitum access to food and water. All animal experiments were performed with the approval of the Pusan National University Institutional Animal Care and Use Committee (PNU-IACUC) and conformed to the guidelines issued by the Animal Care committee of the Institute of Laboratory Animal Resources of Pusan National University (PNU-2019-2200).

### 2.2. Reagents

Macrophage colony-stimulating factor (M-CSF) was purchased from Peprotech (Rocky Hill, NJ, USA) and receptor activator of nuclear factor kappa-Β ligand (RANKL) from ProsPec (Rehovot, Israel). GRP and RC-3095 were purchased from Sigma (St. Louis, MO, USA). The antibodies against GRP and GRPR were from Abcam (Cambridge, UK) and Santa Cruz Biotechnology (Santa Cruz, CA, USA), respectively. The antibody against β-actin was from Cell Signaling (Danvers, MA, USA). Mayer’s hematoxylin was from Dako (Glostrup, Denmark). HRP-conjugated secondary antibody and DAB were from Vector Labs (Burlingame, CA, USA). Lipopolysaccharide (LPS) from *P. gingivalis* was purchased from Sigma Aldrich (St. Louis, MO, USA).

### 2.3. Cell Lines and Cultures

Bone marrow-derived macrophages (BMMs) of five-week-old female ICR mice were used as precursor cells for osteoclasts, as previously described [12]. Mice were euthanized with carbon dioxide. Isolated BMMs were cultured in α-minimum essential medium (α-MEM; Welgene Inc., Daegu, Republic of Korea) supplemented with 10% FBS (Gibco, Co Dublin, Ireland) and 1% penicillin-streptomycin (Thermo Fisher Scientific, Waltham, MA, USA) in the presence of M-CSF (30 ng/mL) for one day and differentiated into osteoclasts using RANKL (100 ng/mL) for three to nine days depending on the experimental condition. Culture media was changed every 2 days. Subsequently, BMMs were fixed with 4% formaldehyde and stained using tartrate-resistant acid phosphatase (TRAP) kit (Sigma Aldrich, St. Louis, MO, USA). The number of TRAP-positive (TRAP+) multinucleated cells (MNCs) with three or more nuclei were counted for evaluation. Bone resorption activity of BMMs was determined using a bone resorption assay kit (Cosmo Bio. Co. Ltd., Tokyo, Japan), according to the manufacturer’s protocol. Briefly, BMMs were seeded in fluoresceinated calcium phosphate-coated microplate and cultured with M-CSF and RANKL in the presence or absence of GRP. After 8 days, pit number and area were analyzed using Image J software after removing the cells from each group in triplicate.

Mouse primary epithelial cells were isolated from buccal mucosa of C57BL/6 wild type mice at 6 weeks of age euthanized with CO_2_, as previously described [13,14], for gene expression analyses, coculture, and conditioned medium experiments. After dissection, the tissues were washed several times with penicillin/streptomycin in PBS and cut into small pieces with razor blades. The epithelial fragments were treated with 1.5 U/mL dispase (Stemcell Technologies, Vancouver, Canada) at 37 °C for 1 h and separated from the lamina propria with forceps. After trypsinization, epithelial cells were filtered through 40 µm cell strainer and cultured with CnT-Prime media (CELLnTEC, Bern, Switzerland).

HaCaT cells were purchased from Thermo Fisher Scientific and cultured in Dulbecco’s modified Eagle medium (D-MEM; Gibco, Co Dublin, Ireland) for gene expression analyses.

### 2.4. Cell Proliferation Assay

Cell proliferation of BMMs treated with either GRP or RC-3095 was evaluated through Days 0 to 3 using Cell Counting Kit-8 (CCK-8; Dojindo Laboratories, Kumamoto, Japan). The assay was carried out according to the manufacturer’s protocol. Colorimetric changes were measured at 450 nm using a Benchmark microplate reader (Bio-Rad Laboratories, Hercules, CA, USA).

### 2.5. Real-Time Quantitative Polymerase Chain Reaction (PCR) Analysis

RNA was extracted using a RNeasy Mini Kit (Qiagen, Germantown, MD, USA) and reverse transcription was performed using a T100 Thermal Cycler (Bio-Rad, Hercules, California, USA). Quantitative analyses of gene expressions were performed with 2 µg of cDNAs amplified with SYBR green PCR master mix (Applied Biosystems) in a MicroAmp optical tube (Applied Biosystems) for 40 cycles of denaturation (15 sec) at 95 °C and amplification (60 sec) at 60 °C in AB7500 instruments (Applied Biosystems). The primer sets used were as follows: *Tmsf4* forward, 5′-GGGTGCTGTTTGCCGCTG-3′; *Tmsf4* reverse, 5′-CGACTCCTTGGG TTCCTTGCT-3′; *Trap* forward, 5′-CGACCATTGTTAGCCACATA CG-3′; *Trap* reverse, 5′-TCGTCCTGAAGATACTGCAGGTT-3′; *Cathepsin K* (*Ctk*) forward, 5′-AGGCGGCTATA TGACCACTG-3′; *Ctk* reverse, 5′-CCGAGCCAAGA GAGCATATC-3′; *Grp* forward, 5′-ATGAATCCCCGTCCCTGTATG-3′; *Grp* reverse, 5′-AGGAGGTCCAGCAAA TCCCTT-3′; *Grpr* forward, 5′-AATTGGCTGCAAACTGATCCC-3′; *Grpr* reverse, 5′- TGGCCG TACAATGGCTTTGT-3′; *β-actin* forward, 5′-TCTGGC ACCACACCTTCTAC -3′; *β-actin* reverse, 5′-TACGACCAGAGGCATACAGG-3′; human *Grp* forward, 5′- GGGGCACTTAAT GGGGAAAAAG-3′; human *Grp* reverse, 5′-GCAGCTTCTTCCCACCTGATG-3′; human *Grpr* forward, 5′-CTGATCCAGAGTGCTTACAA-3′; human *Grpr* reverse, 5′-CGGTACAGGT AGATGACATGA-3′; human *β-actin* forward, 5′-ACTCTTCCAGCCTTCCTTCC-3′; human *β-actin* reverse, 5′-TGTTGGCG TACAGGTCTTTG-3′. All reactions were run in triplicate.

### 2.6. Western Blot Analysis

Cell lysates were collected using radioimmunoprecipitation assay (RIPA) lysis buffer (50 mM Tris pH 8.0, 150 mM NaCl, 0.5% sodium deoxycholate, 1 mM EGTA, 1% Triton X-100, 10 mM NaF, and complete protease inhibitor cocktail). Then, 20 μg of protein lysates were separated using SDS-PAGE (10% or 15% gel). After transferring the separated protein bands onto nitrocellulose membranes, the membranes were blocked with 5% skim milk for 1 h. The primary antibodies against GRP and GRPR were diluted to 1:1000 and β-actin to 1:10,000. The secondary HRP-conjugated antibodies against mouse or rabbit were diluted to 1:5000. Proteins were visualized by using chemiluminescence ECL Prime Western Blotting Detection Reagent (Amersham, Buckinghamshire, UK).

### 2.7. siRNA Transfection

BMMs were transfected with 20 nM of control or *Grpr* siRNAs (Santa Cruz Biotechnology, Inc, Santa Cruz, CA, USA) using jetPRIME transfection reagent (Polyplus transfection, Illkirch, France). After 24 h, media was replaced with fresh α-MEM and cells were cultured, as described above. The knockdown efficiency was examined by quantitative polymerase chain reaction (PCR).

### 2.8. Experimental Periodontitis in Mice

The 5-0 silk ligatures (AILEE Inc., Busan, Republic of Korea) were tied subgingivally around the left maxillary second molar, as previously described [15] under general anesthesia induced by intraperitoneal injection of 400 mg/kg of 2,2,2-tribromoethanol (Avertin, Sigma-Aldrich, St. Louis, MO, USA). The contralateral tooth was unligated as a baseline control. Ligatures were maintained in place until Day 7 and mice were sacrificed on Day 14 for micro computerized tomography (micro-CT), histological, and immunohistological analyses. Mice were perfused with 4% paraformaldehyde via the left ventricle for 5 min. Then, the maxillas was excised, fixed with 4% paraformaldehyde, pH 7.4, at 4 °C overnight, and stored in PBS until analyses.

Micro-CT imaging was done in a SMX-90CT instrument (Shimadzu Corp., Kyoto, Japan) using 90 kV, 100 mA, and 360° of angular range as scanning parameters. The images were reconstructed using inspeXio SMX-90CT software (Shimadzu Corp., Kyoto, Japan). All images were oriented to parallel the cementoenamel junction (CEJ) from the first and the second molar to the horizontal axis.

For histological analysis, maxillas were fixed and decalcified with 5% ethylenediaminetetraacetic acid (EDTA) and 4% sucrose in PBS, for 2 weeks, at 4 °C. Then, maxillas were embedded in OCT compound and 10 µm thick sections were prepared for staining with either TRAP or rabbit monoclonal anti-GRP antibody. The sections for the latter were incubated with HRP-conjugated secondary antibody and DAB substrate. All sections were counterstained with hematoxylin (Dako, Glostrup, Denmark) and images were acquired by Nikon Eclipse Ts2 (Nikon, Tokyo, Japan).

### 2.9. Human Gingival Tissue Samples

Gingival tissue samples were obtained from premolar of patients (13 clinically healthy gingivae and 13 periodontitis-affected gingivae from 9 patients) during routine periodontal flap surgery at Pusan National University Dental Hospital (PNUDH, IRB number PNUDH-2020-002). All patients were non-smoking and did not have untreated systemic or metabolic diseases at the time of sample collection. Periodontitis was diagnosed by a pocket depth ≥4 mm, attachment loss ≥3 mm, and positive for bleeding upon probing. For immunostaining, samples were washed with sterile saline and fixed in 10% formalin solution. Then, they were embedded in paraffin and sectioned to 10 µm thickness for immunostaining with GRP. For RNA isolation, gingival biopsies were stored in RNAlater solution (Thermo Fisher Scientific, Waltham, MA, USA) at −70 °C after washing briefly with saline. RNA was isolated from the frozen tissues using a mirVana RNA isolation kit (Thermo Fisher Scientific, Waltham, MA, USA).

### 2.10. Bacterial Strain and Growth Conditions

*P. gingivalis* (ATCC 33277) was grown in tryptic soy broth supplemented with 1 µg/mL hemin (Sigma-Aldrich, St. Louis, MO, USA). The broth was sterilized by autoclaving, and 1 µg/mL vitamin K (Sigma-Aldrich, St. Louis, MO, USA) was added after cooling. Bacterial culture was performed under strict anaerobic conditions (5% H_2_, 5% CO_2_, 90% N_2_) at 37 °C. Colony forming units (CFU) were estimated by optical density reading taken at 660 nm. Cultured were pelleted at 3000 rpm, for 15 min, at room temperature and resuspended in sterile PBS before use.

### 2.11. Coculture

ECs and BMMs were cocultured using Transwell inserts with 3 µm pore filters (Corning, NY, USA). BMMs were seeded in the lower chamber of a 24-well plate with M-CSF and cultured for two days. ECs (passage 1) were seeded in the Transwell inserts. After 2 days, ECs were inoculated with *P. gingivalis* for 3 h. Twenty-four hours after inoculation, the inserts with ECs were moved to the wells containing BMMs to establish the coculture system. The culture was maintained for 9 days and media were refreshed every three days. BMMs were stained for TRAP activity.

### 2.12. Conditioned Medium Treatment

Conditioned medium (CM) from ECs were obtained 24 h after stimulation with either *P. gingivalis* LPS or live *P. gingivalis*. BMMs were seeded in 48-well plates and allowed to proliferate for 24 h. BMMs’ media was substituted by 2:8 CM/fresh medium and BMMs were cultured to differentiate. After 8 days, cells were fixed for TRAP staining.

### 2.13. Statistical Analysis

Statistical analyses were performed using ANOVA in excel. Values were considered statistically significant at *p* < 0.05. All data are presented as mean ± SD. Results are representative of at least three independent experiments.

## 3. Results

### 3.1. Gastrin-Releasing Peptide (GRP) Promtoes Osteoclast Differentiation

**Figure 1:**
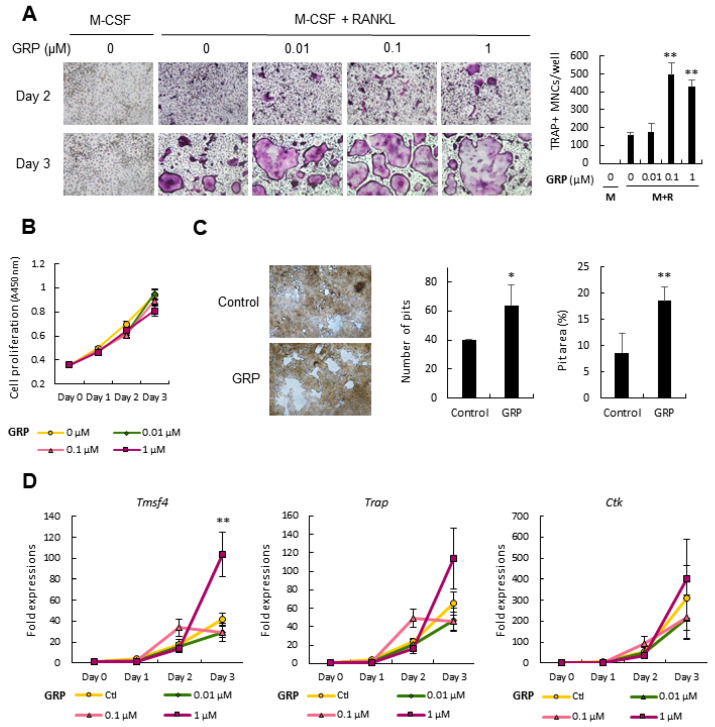
Gastrin-releasing peptide (GRP) treatment stimulates osteoclastogenesis. (**A**) Bone marrow-derived macrophages (BMMs) were cultured with macrophage colony-stimulating factor (M-CSF) and receptor activator of nuclear factor kappa-Β ligand (RANKL) in the presence of increasing concentrations of GRP. The number of tartrate-resistant acid phosphatase positive (TRAP+) multinucleated cells (MNCs) were quantified on Day 3; (**B**) Cell Counting Kit-8 (CCK-8) assay showed that there are no significant differences in the proliferation of BMMs between groups treated with different concentrations of GRP; (**C**) On Day 8, resorption activity of osteoclasts in the presence or absence of 0.1 μM GRP was analyzed by quantifying the number of pits the percentage of eroded area; (**D**) Quantitative real-time polymerase chain reaction (PCR) analyses of molecular markers of osteoclast fusion (*Tmsf4*) and osteoclast differentiation (*Trap* and *Ctk*) through Days 0 to 3. Scale bars, 100 µm. Magnification, ×100. Quantitative data are presented as mean ± SD. * *p* < 0.05 and ** *p* < 0.01 by ANOVA test. Asterisk shows a significant difference from the control or Day 0 group.

To address the role of GRP in osteoclast differentiation, we stimulated murine bone marrow-derived macrophages (BMMs) with RANKL in the presence or absence of GRP for three days. BMMs are widely used mouse primary osteoclast precursors which differentiate into bone-resorbing osteoclasts upon RANKL stimulation. GRP induced osteoclast differentiation in a dose-dependent manner, as shown by the increased number of TRAP-positive multinucleated osteoclasts on Day 3 (Figure 1A). Next, GRP’s effect on the proliferation of BMMs was tested and there was no significant difference in the proliferation of BMMs treated with different concentrations of GRP (Figure 1B). To determine the effect of GRP on osteoclasts’ resorbing activity, BMMs were cultured on fluoresceinated calcium phosphate-coated microplate. In agreement with TRAP staining results, GRP-treated BMMs displayed increased resorption pit number and area (%) on Day 8 of culture (Figure 1C). Furthermore, we sought to examine the molecular markers of osteoclast differentiation after GRP treatment. Among several genes, only the expression of *Tmsf4*, the gene coding DC-STAMP essential for osteoclast fusion, was significantly induced by 1 μM GRP treatment (Figure 1D), although the expression of differentiation marker genes, such as *Trap* and *Ctk*, showed an increasing trend at higher GRP concentrations.

### 3.2. Pro-Osteoclastogenic Effect of GRP Can Be Blocked by Antagonizing or Silencing Gastrin-Releasing Peptide Receptor (GRPR)

**Figure 2:**
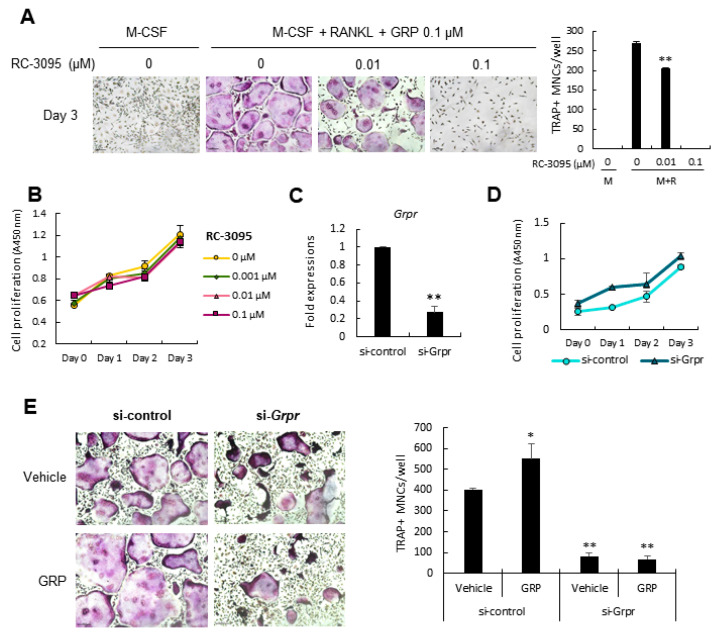
Antagonizing or silencing *Grpr* abolishes the pro-osteoclastogenic effect of GRP. (**A**) BMMs were cultured with increasing concentrations of gastrin-releasing peptide receptor (GRPR) antagonist RC-3095. The number of TRAP+ MNCs were quantified on Day 3; (**B**) CCK-8 assay showed that RC-3095 did not affect BMMs proliferation; (**C**) The efficiency (~70%) of *Grpr* gene knockdown in BMMs using siRNA transfection was confirmed by quantitative PCR; (**D**) CCK-8 assay showed that there are no significant differences in the proliferation of BMMs between si-control and si-*Grpr*; (**E**) On Day 8, a significant decrease in osteoclastogenesis is observed in the si-*Grpr* group as compared with the si-control group. Scale bars, 100 μm. Magnification, ×100. Quantitative data are presented as mean ± SD. * *p* < 0.05 and ** *p* < 0.01 by ANOVA test. Asterisk shows a significant difference from the control or vehicle group.

To examine if GRP/GRPR signaling had an immediate effect on osteoclast differentiation, we exposed BMMs to RC-3095, a GRPR antagonist, along with GRP. After three days, we observed that TRAP-positive osteoclast formation was decreased in a dose-dependent manner (Figure 2A), which suggests that GRP signals via GRPR receptor subtype to regulate osteoclast differentiation. CCK-8 assay revealed that RC-3095 does not significantly affect the proliferation of BMMs (Figure 2B). Next, we silenced *Grpr* in BMMs using siRNA transfection. Gene knockdown was validated using qPCR (Figure 2C), and CCK-8 assay showed that the transfection did not affect the proliferation of BMMs (Figure 2D). Interestingly, TRAP staining showed that *Grpr* silencing strongly decreased the number of osteoclasts and adding GRP did not revert this effect (Figure 2E). Taken together, these results show that antagonizing or silencing *Grpr* abrogates the pro-osteoclastogenic effect of GRP.

### 3.3. Grp and Grpr Expressions in Bone Marrow-Derived Macrophages (BMMs) Are Downregulated during Osteoclcastogenesis

**Figure 3:**
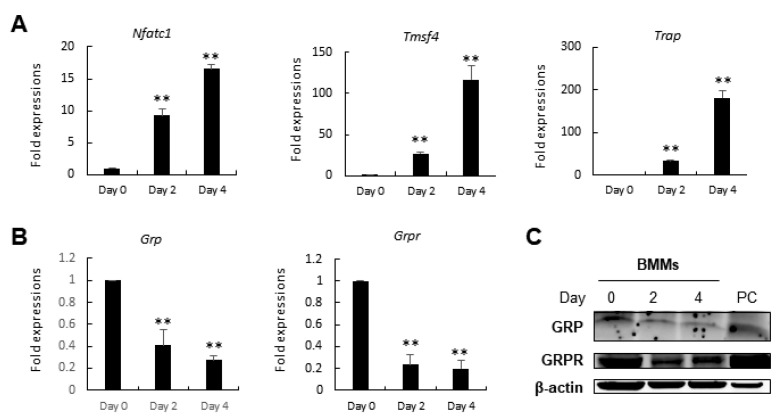
*Grp* and *Grpr* expressions are downregulated during osteoclastogenesis. (**A**) Expression of markers of osteoclastogenic differentiation increased in BMMs during osteoclastogenesis while (**B**) *Grp* and *Grpr* expressions decreased; (**C**) Protein analysis confirms the downregulation of GRP and GRPR during osteoclastogenesis. 3T3L1 cells at Day 7 were used as a positive control (PC). Quantitative data are presented as mean ± SD. ** *p* < 0.01 by ANOVA test. Asterisk shows a significant difference from Day 0 group.

Next, we examined the expression of *Grp* and *Grpr* during osteoclastogenesis. While the expression of *Nfatc1*, a key gene for osteoclast differentiation, *Tmsf4*, and *Trap* increased during osteoclastogenesis (Figure 3A), the expression of *Grp* and *Grpr* was significantly downregulated (Figure 3B). Moreover, there was a similar reduction in the protein levels of GRP and GRPR during osteoclast differentiation (Figure 3C). These results suggest that there may exist another source of GRP in pathologic bone resorption.

### 3.4. GRP Expression Is Upregulated in Mouse and Human Periodontitis Tissues

**Figure 4:**
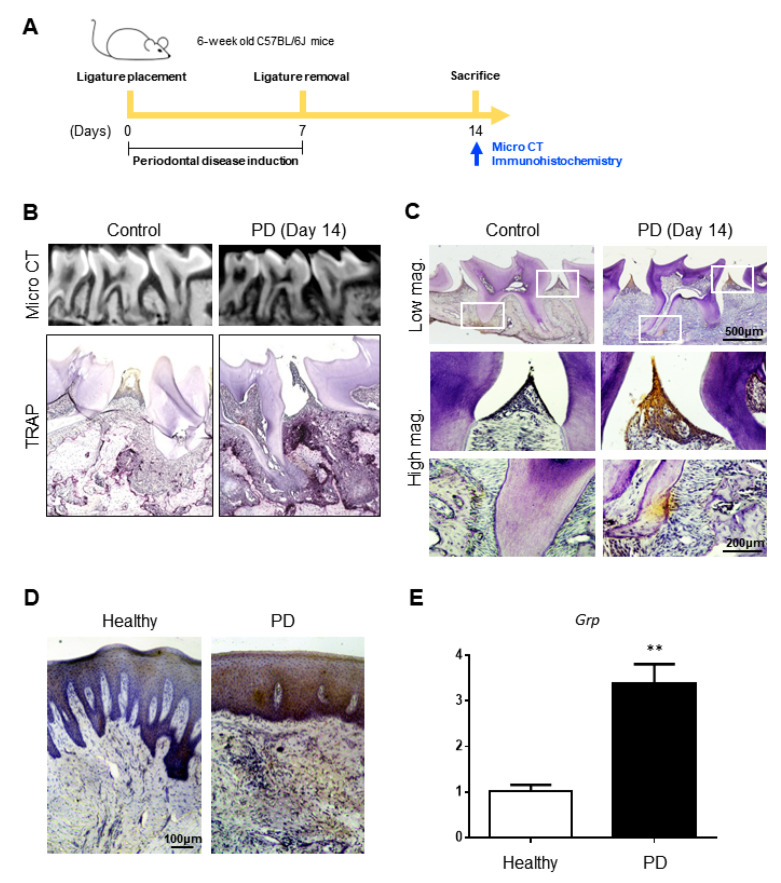
Periodontitis-associated bone resorption and GRP accumulation. (**A**) Schematic diagram depicting the timing of ligation and sample collection of ligature-induced periodontitis model in mice; (**B**) Resorption of alveolar bone around the teeth (as indicated by arrows) and the root bifurcation area (as indicated by an asterisk) can be observed in the maxillary section of the periodontitis (PD) group. TRAP staining displayed higher number of TRAP+ cells in the alveolar bone between the maxillary first and second molar (as indicated by an arrow) 14 days after ligation. Histological staining of anti-GRP antibody detected with peroxidase substrate DAB (brown) is prominent in the epithelium of (**C**) mouse and (**D**) human gingival tissue samples in PD groups; (**E**) Quantitative PCR analysis show upregulation of *Grp* expression in the gingival samples of PD patients. Scale bars as indicated, ×40 for low magnification, and ×100 for high magnification. Quantitative data are presented as mean ± SD. ** *p* < 0.01 by ANOVA test. Asterisk shows a significant difference from the healthy group.

On the basis of the results of previous experiments, we proceeded to investigate the expression pattern of GRP in mouse and human periodontitis. Gingival tissue samples from mouse model and patients diagnosed with periodontitis were examined for GRP expression by histological staining. An experimental in vivo study was carried out using ligature-induced periodontitis model, as described in the schematic diagram (Figure 4A). According to the micro-CT analysis, the ligation successfully induced periodontitis in mice by causing bone height reduction around the teeth including the alveolar bone and the root bifurcation area (Figure 4B). TRAP staining of mouse maxilla two weeks after ligation showed increased osteoclast formation at the alveolar crest (Figure 4B). Intriguingly, GRP-positive cells were mostly located at the oral epithelium of samples from experimental periodontitis model (Figure 4C). Furthermore, GRP staining was predominantly observed in the epithelium of the periodontal lesion in patients (Figure 4D). Quantitative PCR results also indicated that *Grp* expression levels in samples from patients with periodontitis are at least three-fold higher than in healthy gingiva samples (Figure 4E). Collectively, analyses on the GRP profiles in periodontitis-affected tissues delineated the abnormal expressions of GRP in epithelial cells (ECs).

### 3.5. Lipopolysaccharides (LPS)- or P. gingivalis-Stimulated ECs Induce the Osteoclastic Differentiation of Cocultured BMMs

**Figure 5:**
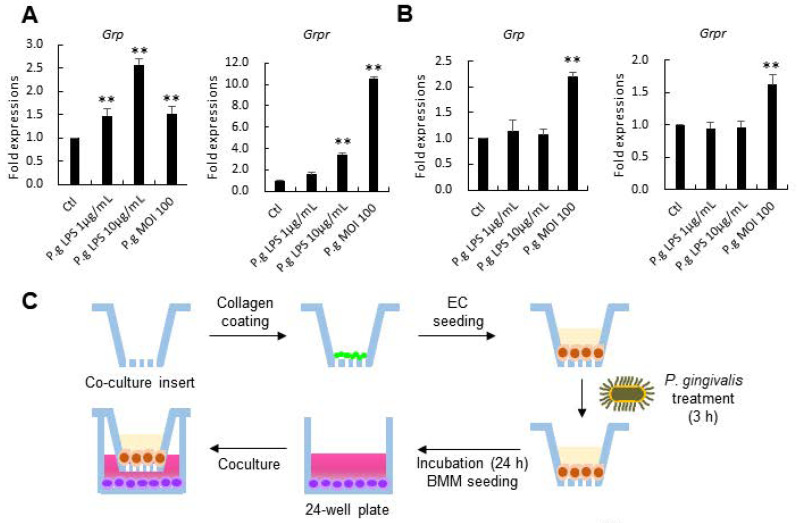

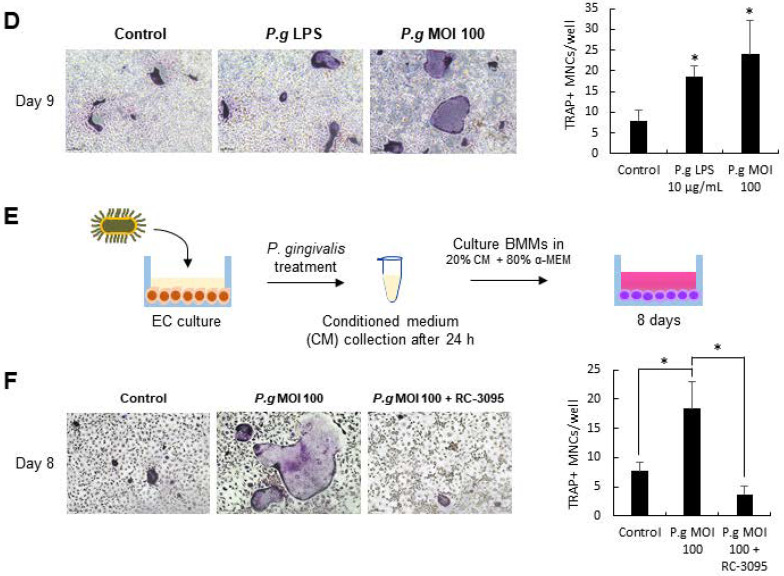
*P*. *gingivalis*-stimulated epithelial cells (ECs) can induce the differentiation of BMM. *Grp* and *Grpr* expressions in (**A**) mouse ECs and (**B**) HaCaTs; (**C**) Experimental procedure of coculture using ECs and BMMs in non-contact Transwell in the presence of 30 ng/mL M-CSF and 50 ng/mL RANKL to induce osteoclastogenesis; (**D**) TRAP+ osteoclast formation was promoted in cocultures with *P. gingivalis*-treated ECs; (**E**) Preparation of conditioned medium (CM) from *P. gingivalis*-treated (1 or 10 μg/mL LPS and MOI 100) ECs; (**F**) BMMs cultured in 20% CM from *P.* gingivalis-inoculated ECs generated higher number of osteoclasts than the control. Scale bars, 100 µm. Magnification, ×100. Quantitative data are presented as mean ± SD. * *p* < 0.05 and ** *p* < 0.01 by ANOVA test. Asterisk shows a significant difference from the control group unless indicated otherwise.

In the following analyses, we focused on ECs to explore their potential supporting effect on the osteoclastogenesis of BMMs. ECs were stimulated by LPS of *P. gingivalis* (1 or 10 μg/mL) or inoculated with live *P. gingivalis* at multiplicity of infection (MOI) of 100, for 24 h. Consistent with results from Figure 4, LPS stimulation and *P. gingivalis* inoculation increased the expression of *Grp* and *Grpr* in mouse epithelial cells (Figure 5A). In HaCaT, *Grp* and *Grpr* expressions were upregulated by ~2 folds upon inoculation with *P. gingivalis* (Figure 5B). HaCaT is an immortal yet nontumorigenic cell line frequently employed to multilayered cell culture models of the oral cavity. Next, to gain insight on how BMMs were regulated by ECs in the periodontal microenvironment, a non-contact coculture model using Transwell insert was designed, as shown in the schematic diagram (Figure 5C). ECs were stimulated by *P. gingivalis* LPS (10 μg/mL) or inoculated with live *P. gingivalis* (MOI 100) for 24 h and cocultured with BMMs to test if GRP produced by ECs could directly mediate the differentiation of BMMs. BMMs cocultured with unstimulated ECs were regarded as control groups. TRAP staining results showed that coculture with LPS- or *P. gingivalis*-stimulated ECs effectively promoted the differentiation of BMMs into osteoclasts (Figure 5D). These findings suggest that paracrine effects of ECs on the osteoclastogenesis potential of BMMs were persistent and relatively stable. Finally, BMMs were cultured with the conditioned medium (CM) from ECs to confirm the influence of *P. gingivalis* on the pro-osteoclastogenic supporting activity of ECs (Figure 5E). To discern the specificity of GRP in ECs-mediated osteoclastogenesis, RC-3095 was added to the CM of each group. Only the CM from *P. gingivalis*-inoculated ECs in 2:8 concentration stimulated the formation of TRAP-positive osteoclasts (Figure 5F). In addition, abolishing GRP’s effect via RC-3095 inhibited the ability of the CM to induce osteoclast differentiation. These results suggested that ECs may be able to regulate osteoclastogenesis via a mechanism that involves soluble GRP.

## 4. Discussion

In this study, we report a unique function for GRP and its receptor GRPR in manifesting increased differentiation of BMMs. We found that GRP strongly potentiated the formation of mature osteoclasts, but we do not yet know the molecular mechanism of GRP-specific ability to stimulate osteoclastogenesis. Our next approach will focus on identification of the signaling pathways mediated by GRP.

In the regulation of the immune response, GRP is known to play a role by acting directly on GRPR-expressing immune cells. GRP signaling is initiated by Gaq and Ga12/13 heterotrimeric G proteins [5]. Gaq signaling leads to the activation of mitogen-activated protein kinase (MAPK) and extracellular signal-regulated kinase (ERK), while Ga12/13 leads to the activation of c-Jun N-terminal kinase (JNK) and p38 [16]. Interestingly, protein kinase signaling pathways activated during osteoclastogenesis include JNK, p38, ERK, Src, and inhibitor of NF-κB kinase (IKK) [17]. Our Western blot analysis showed that GRP did not induce the protein levels of NFATc1 (data not shown), a master transcription factor induced by aforementioned signaling pathways for the terminal differentiation of osteoclasts. The reason why GRP shows no significant effect on NFATc1 during osteoclastogenesis is not clear, and further studies are needed in the future.

Meanwhile, the real-time PCR results indicated that GRP treatment significantly upregulated the expression of *Tmsf4*, the gene encoding DC-STAMP in BMMs, and we suspect that GRP promotes osteoclastogenesis by upregulating the expression of DC-STAMP. DC-STAMP is a molecule essential for the fusion of mononuclear osteoclasts and formation of fully functional osteoclasts [18]. The evidence suggests that the expression of DC-STAMP is regulated by NFATc1 [19], but there are other osteoclastogenesis-regulating pathways that may be responsible for the induction of DC-STAMP. It has been reported that B cell lymphoma 6 (Bcl6), a transcriptional repressor highly expressed in osteoclast progenitors but significantly downregulated during osteoclastogenesis by RANKL, directly binds to *Tmsf4* promotor [20]. Additional studies are underway to elucidate the molecular mechanisms involving GRP in osteoclast differentiation and fusion.

Previous studies have discovered that periodontitis and other orofacial inflammatory disorders may be modulated by imbalances in certain neuropeptides [21]. Among the neuropeptides, upregulation of substance P (SP) in the gingival crevicular fluid (GCF) of patients with periodontitis has been observed, and its levels have been found to be decreased after treatment [22]. In GCF of periodontitis-affected sites, SP was able to augment cytokine production and act as a pro-inflammatory mediator by limiting the production of TGF-β by LPS-activated macrophages [23]. The role of GRP, as a neuropeptide in exerting a stimulatory effect on a complex regulatory network of inflammatory mediators and immune cells during periodontitis, is presumable, but understanding the neurogenic component of periodontal disease is challenging because changes in neuropeptide levels are only part of a cascade of chemical activity [21].

More importantly, *Grp* and *Grpr* expressions have been enhanced in in vitro and ex vivo models of calcification, and GRPR antagonist treatment attenuated GRP’s effect on phosphate-induced vascular calcification [10]. To date, many studies have confirmed the association between bone loss and vascular calcification, and these seemingly unrelated conditions share common pathogenetic mechanisms involving bone morphogenetic proteins, RANKL, matrix Gla protein, cathepsins, and vitamin K [24]. We hypothesized that GRP might be a factor affecting bone loss in periodontitis and set out to investigate changes in GRP expressions in experimental periodontitis models. We successfully demonstrated pro-osteoclastogenic changes of BMMs after GRP treatment and strong GRP immunostaining in the oral epithelium of periodontal tissues from in vivo mouse model and patients.

To validate our results observed in vivo, *P. gingivalis* was used for succeeding in vitro experiments. *P. gingivalis* inoculation presents a convenient and quick experimental model to stimulate infection-triggered inflammation. *P. gingivalis* is a Gram-negative anaerobe considered to be one of the keystone pathogens in the initiation of periodontitis [25,26]. It acts through virulence factors such as LPS, fimbriae, and gingipains to hijack the host immune response, leading to sustained inflammation and, subsequent, tissue destruction at local as well as distant sites [27]. In the periodontal wound environment, a variety of cell types, such as epithelial cells, fibroblasts, osteoblasts, and immune cells, are present and activated through multiple signaling systems following wounding [28,29].

Gingival ECs are the first line of host defense as they function as a physical barrier against invading pathogens. Recent studies have demonstrated that *P. gingivalis* can invade ECs to activate resident periodontal tissue cells which, in turn, affects cell proliferation, differentiation, and migration of precursor immune cells into the wound environment [30,31]. These responses lead to the production of various inflammatory and immune mediators that contribute to the destruction of tissue components including bone [30]. A recent study showed that oral bacteria and their components stimulated periodontal ligament cells, which, in turn, produced IL-6 that induced the accumulation of T helper 17 (T_H_17) cells and exFoxp3 T_H_17 cells in the oral mucosa [32]. These are a subset of T cells with pro-osteoclastogenic properties. However, this experiment was the first to identify some level of cell-to-cell communication between ECs and macrophages in promoting osteoclast differentiation in the periodontal microenvironment. Our present data suggest that the abnormal GRP in periodontitis-affected ECs is linked to pathological activation of osteoclastogenesis. It also indicates that communication exists between the vasculature and bone and GRP may act as a link between periodontal disease and cardiovascular disease.

**Figure 6:**
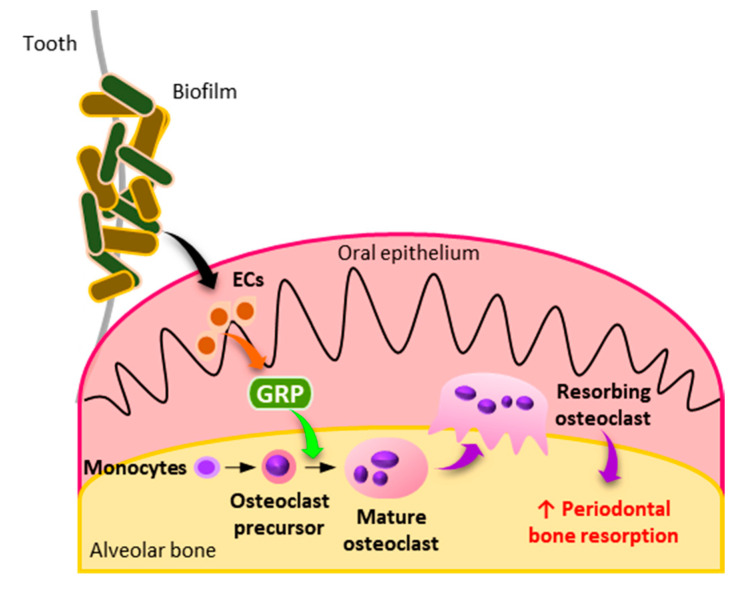
Illustration of this study’s results. Under inflammatory conditions during periodontitis, ECs release GRP which binds to GRPR on BMMs. This leads to the activation of GRPR downstream signaling and, subsequently, promotes osteoclastogenesis, resulting in enhanced periodontal bone resorption.

In conclusion, we show that GRP stimulates osteoclast differentiation via GRPR signaling (Figure 6). Moreover, we provide evidence that GRP secreted by ECs in the periodontal microenvironment may be a regulator of osteoclastogenesis. These findings provide novel insight into the molecular mechanisms how BMMs differentiate and contribute to the progression of periodontitis.

## Data Availability

The data presented in this study are available within this article.
